# A Transcriptomic Analysis of Phenotypic Plasticity in *Crassostrea virginica* Larvae under Experimental Acidification

**DOI:** 10.3390/genes13091529

**Published:** 2022-08-25

**Authors:** Michelle Barbosa, Caroline Schwaner, Emmanuelle Pales Espinosa, Bassem Allam

**Affiliations:** School of Marine and Atmospheric Sciences, Sony Brook University, Stony Brook, NY 11790, USA

**Keywords:** oyster, ocean acidification, RNASeq, acclimation

## Abstract

Ocean acidification (OA) is a major threat to marine calcifiers, and little is known regarding acclimation to OA in bivalves. This study combined physiological assays with next-generation sequencing to assess the potential for recovery from and acclimation to OA in the eastern oyster (*Crassostrea virginica*) and identify molecular mechanisms associated with resilience. In a reciprocal transplant experiment, larvae transplanted from elevated *p*CO_2_ (~1400 ppm) to ambient *p*CO_2_ (~350 ppm) demonstrated significantly lower mortality and larger size post-transplant than oysters remaining under elevated *p*CO_2_ and had similar mortality compared to those remaining in ambient conditions. The recovery after transplantation to ambient conditions demonstrates the ability for larvae to rebound and suggests phenotypic plasticity and acclimation. Transcriptomic analysis supported this hypothesis as genes were differentially regulated under OA stress. Transcriptomic profiles of transplanted and non-transplanted larvae terminating in the same final *p*CO_2_ converged, further supporting the idea that acclimation underlies resilience. The functions of differentially expressed genes included cell differentiation, development, biomineralization, ion exchange, and immunity. Results suggest acclimation as a mode of resilience to OA. In addition, the identification of genes associated with resilience can serve as a valuable resource for the aquaculture industry, as these could enable marker-assisted selection of OA-resilient stocks.

## 1. Introduction

The Earth’s climate is changing at an unprecedented rate. Ocean acidification (OA), the reduction of seawater pH from increasing partial pressure of carbon dioxide (*p*CO_2_), has rapidly gained recognition as a major threat for marine organisms and ecosystems. Since the 19th century, carbon dioxide (CO_2_) concentrations have increased from 280 parts per million (ppm) to over 400 ppm with a 0.1 decrease in seawater pH, and the Intergovernmental Panel on Climate Change (IPCC) predicts concentrations will reach 800–1000 ppm by the end of the century [1]. With the predicted increase in atmospheric CO_2_, the pH of seawater is predicted to further decrease from 8.2 to 7.8. In coastal areas, acidification is exacerbated by land-based sources of pollution that foster intense algal blooms. For example, coastal waterways around Long Island, NY, have already reached low pH conditions predicted for the end of the century (pH < 7.4) in the late summer, partially as a result of these blooms and the resulting microbial respiration [2]. Furthermore, the Long Island Sound is predicted to reach the mean annual chemical threshold for disruption of calcification and development of larval bivalves as early as 2071–2099 [3].

Increasing CO_2_ in seawater reduces the availability of calcium carbonate (CaCO_3_), which limits the deposition of material for organisms with CaCO_3_ skeletons or shells and can cause the dissolution of shell material [4,5]. Poor development of shells can affect normal physiological function, weaken hosts to pathogens, or increase vulnerability to predation. In the last ten years, a number of studies have documented the adverse effects of OA on the metabolic activity of marine calcifiers. Though not all taxa respond in the same manner to elevated *p*CO_2_, many bivalve mollusks exhibit metabolic depression [6] under experimental OA conditions. This depression manifests as reduced growth [7,8], survival [9], development [10], immunity [11,12,13], and reproduction [14]. Numerous studies have demonstrated that larval stages are most susceptible to the adverse effects of OA [13,15,16].

The eastern oyster (*Crassostrea virginica*) has been identified as a potentially vulnerable species under future climate conditions [17,18,19]. Oysters are important both ecologically and economically and the loss of oysters could have profound effects on global ecosystems and economies. Fortunately, some individuals are expected to persist in the face of climate change; however, what confers resilience in these individuals is relatively unknown. To date, our understanding of the effects of OA on bivalves has almost exclusively focused on the physiological effects low pH will have on animal health and survival; this somewhat superficial understanding does not probe the processes that enable bivalves to survive and tolerate OA. In addition, the ability for oysters exposed to OA to “recover” physiological function in normal pH conditions is also unknown. This knowledge could be critical to determining the extent to which bivalve stocks are at risk in future ocean conditions.

Recent studies have begun to explore the means for resilience and potential for recovery in calcifying species exposed to OA [20,21,22]. Phenotypic plasticity, the ability of an organism of one genotype to produce multiple phenotypes under different environmental conditions [23], has been suggested as a mechanism to tolerate OA [6,24,25,26]. However, whether the described effects of OA on the physiology of these organisms is the result of phenotypic plasticity and is reversible is unknown for many species, including the eastern oyster. Of the few studies examining plasticity under OA, some make use of a reciprocal transplant design [6,13,27,28]. The benefit of the reciprocal transplant design is that it allows for the identification of plasticity with simple physiological assays that examine recovery, while also enabling the use of next-generation sequencing (NGS) technologies that can identify underlying molecular processes. If individuals retain some plasticity for variable pH environments and acclimation is an important mechanism for resilience, animals may recover once transplanted from OA to ambient conditions. If an individual’s genotype exhibits plasticity, then they may tolerate a wider range of suboptimal pH at the expense of normal physiological function. Thus, returning oysters to optimal conditions after OA stress may result in a rebound in growth and survival.

Rebound potential has been demonstrated in two species of tropical sea urchins, *Lytechinus variegatus* and *Echinometra luccunter*, in which OA-exposed animals were re-exposed to normal conditions and partially recovered immune functions [29]. In *Pseudocalanus acuspes*, a calanoid copepod, reciprocal transplant from elevated to ambient *p*CO_2_ conditions resulted in higher egg production rates and larger clutch sizes at lower *p*CO_2_ [27]. Authors describe these results as suggestive of phenotypic plasticity in the copepod species. Reciprocal transplantation has not been undertaken thus far with larval eastern oysters that were reared in acidified conditions and then transplanted to normal *p*CO_2_ conditions.

Changes in gene expression levels have been suggested as a compensatory response to OA [30]. As gene expression is a highly plastic response to changing environmental conditions [31], assessing changes in expression makes for an excellent choice when studying the effects of OA on bivalves and their potential to acclimate to it. Comparing the relative expression levels between populations under control and stressed conditions can lead to the identification of genes either affected by the stress or vital to the resilience of the organism, or both. A growing number of studies have begun to examine shifts in gene expression in response to OA [12,21,32,33,34,35]. Studies have demonstrated variability in *C. virginica* gene expression associated with changes in pH, with genes exhibiting differential expression at low pH [36]. Studies in other bivalve species have shown differential regulation of genes related to biomineralization and immunity, which has been suggested to function in frontloading the immune system [37]. In the bivalve *Laternula elliptica,* exposure to low pH resulted in upregulation of the enzyme chitin synthase (an enzyme associated with synthesizing shell) [38]. The genes affected under OA and the degree to which the expression of those genes is upregulated or downregulated, as an associated “cost”, appear to be species-specific and age-specific. However, acclimation via gene expression plasticity shows promise as a mechanism for resilience to OA.

Despite the increase in transcriptomic analyses, few studies have examined the changes in gene expression level for *C. virginica* larvae exposed to OA stress and fewer studies have also combined this with physiological assays. By combining RNAseq with the experimental design of the reciprocal transplant experiment (i.e., transplanted treatments), it will be possible to identify acclimation and plasticity if there is convergence of gene expression patterns of non-transplanted and transplanted larvae under the same final *p*CO_2_.

The study described here expands upon the research published for other sensitive species by contributing to understanding whether recovery of the eastern oyster following exposure to OA is possible and whether molecular features underlie resilience. The objective of this study was to evaluate the ability of the eastern oyster to recover from short-term exposure to elevated *p*CO_2_ and to probe the molecular mechanisms associated with this recovery. To do so, we contrasted growth, survivorship, and transcriptomic profiles (RNAseq) between larvae transplanted from OA conditions to ambient conditions (and vice versa) and those kept under stable *p*CO_2_ conditions. *C. virginica* serves as an excellent model for such studies as the early life stage larvae and juveniles are considered to be particularly sensitive to the effects of OA [16]. Results underlined a plastic response to OA and highlighted the ability of oyster larvae to recover from short exposures to OA stress.

## 2. Materials and Methods

### 2.1. Seawater Chemistry

Seawater was collected from either Great South Bay, East Islip, NY (40°42′19.8″ N, 73°11′42.2″ W), or Stony Brook Harbor, NY (40°55′15.1428″ N, 73°9′0.6696″ W), and prepared for use by filtering through a 1 µm filter and sterilizing via UV light. Ambient and elevated *p*CO_2_ conditions were maintained according to the guidelines established by the European Project on Ocean Acidification (EPOCA) [39]. The ambient condition was achieved by bubbling in ambient air to maintain a pH of 8.1 (*p*CO_2_ of ~350 ppm). The elevated condition was achieved by bubbling in a mixture of 5% CO_2_ (balanced in air) and air to maintain a pH of 7.5 (*p*CO_2_ of ~1400 ppm) via the use of a gas proportioner (Cole Parmer^®^ Flowmeter system, multitube frame, Vernon Hills, IL, USA). The target low pH was selected based on end-of-the-century predictions by the IPCC [1] and based on the current pH conditions in the Long Island Sound, which this species inhabits [2]. The pH was monitored daily using an Ohaus ST300 Portable pH Meter (precision of 0.01 pH; Parsippany, NJ, USA). Bubbling of CO_2_ or air began approximately 24 h before the start of the experiment to ensure the correct and stable treatment pH. Samples for dissolved inorganic carbon (DIC) analysis were collected and read using a VINDTA 3D (Versatile Instrument for the Determination of Total inorganic carbon; manufactured by Ludger Mintrop, Marianda, Kiel, Germany) delivery system coupled with a UIC Inc. (Joliet, IL, USA) coulometer (model CM5017O). Bicarbonate standards were used and, for quality assurance, certified reference material was analyzed (provided by Andrew Dickson, Scripps Institution of Oceanography, La Jolla, CA, USA) with a 99.99% recovery during every run. *p*CO_2_, Ω_aragonite_, Ω_calcite_, DIC, CO_3_, and alkalinity were calculated from pH, temperature, and salinity using the seacarb package for R statistical software v3.6.1 (R Core Team, Vienna, Austria) following parameters recommended by [40] with known first and second dissociation constants of carbonic acid in seawater [41]. Seawater chemistry characteristics are provided in Table S1.

### 2.2. Animal Husbandry and Experimental Methods

Adult *C. virginica* were collected from four wild populations to increase the genetic diversity of experimental offspring: Northport, NY (40.9009° N, 73.3432° W); Southampton, NY (40.8840° N 72.4414° W); Mount Sinai Harbor, NY (40.9577° N, 73.0335° W); Wellfleet, MA (41.9305° N, 70.0310° W). Broodstock were conditioned at 18 °C on a diet of live-culture microalgae (*Isochrysis* spp. and *Tetraselmis* spp.) delivered via a drip system over a period of eight weeks. Adults were induced to spawn together via a single thermal shock (i.e., temperature brought up to 28 °C in a period of <15 min) under ambient *p*CO_2_ (~350 ppm, pH 8.1) at Great Atlantic Shellfish Farm (Islip, NY, USA (40.7059° N, 73.1946° W)). Individuals that released eggs were identified as female and placed into a separate sea table consisting only of females for temporary holding and egg collection. Individuals that released sperm were identified as males and were left in the spawning sea table for the collection of sperm. After all individuals had released gametes (7 females and 10 males), sperm was added to the water in which the females had released eggs. The addition of the mixed sperm to the eggs ensured genetic mixing and homogeneity. After allowing sufficient time for fertilization (~1 h), embryos were then collected and immediately (i.e., <2 h post fertilization) exposed to either ambient (*p*CO_2_ of ~350 ppm, pH 8.1) or elevated *p*CO_2_ (*p*CO_2_ of ~1400 ppm, pH 7.5), with four replicates per condition (*n* = 4).

Larvae were initially held in static systems in 43 L vessels at 28 ppt and 20 °C in East Islip, NY (40.7059° N, 73.1946° W), for the first seven days following spawning (*n* = 4 replicate vessels per *p*CO_2_ condition). Larvae were maintained at a stocking density of 10 larvae mL^−1^ (common stocking density in aquaculture [42]) and received a complete (100%) water change every other day. During water changes, animals were passed over a mesh sieve (25 µm), rinsed, and resuspended in 0.2 µm filtered seawater equilibrated to the appropriate pH, temperature, and salinity. Larvae were fed *ad libitum* a mixture of live algae, including *Tisochrysis lutea* and *Isochrysis* spp. grown from semi-continuous culture using f/2 medium.

After the seven days, the larvae were transferred to the Marine Animal Disease Laboratory at Stony Brook University and moved to 18 L vessels (28 ppt, 20 °C, 10 larvae mL^−1^), and larval rearing followed the same procedures described previously. Following the initial seven-day exposure, larvae were transplanted to the alternative *p*CO_2_ condition. Half of the larvae in ambient *p*CO_2_ were transplanted to elevated *p*CO_2_ and half of the elevated *p*CO_2_ larvae were transplanted to ambient *p*CO_2_; the remaining half in each condition were maintained in their original *p*CO_2_ condition to serve as non-transplanted controls. Replicate treatment vessels (eight replicates per *p*CO_2_ condition, including four transplanted and four remaining at the same *p*CO_2_ condition) were randomly assigned to sea tables. Larvae were maintained under pre-transplant and post-transplant conditions for one-week time periods to allow enough time to discern acclimation from an acute stress response and in order to be able to assess changes in physiology.

Larvae were subsampled for viability and growth analysis before transplantation and at the end of the experiment (one-week post-transplantation) by preservation in 1% glutaraldehyde. For viability, larvae were assessed microscopically (range of 350 to 630 larvae per treatment); larvae that lacked internal complexity or had indistinct tissue structure were counted as dead and all other larvae were counted as live prior to preservation. Size of live larvae (length from anterior to posterior edge) was measured using image analysis (ImageJ, NIH, Bethesda, MD, USA) with a range of 190 to 470 larvae per treatment. The remaining larvae were preserved at −80 °C for RNA extraction. Viability and growth data were analyzed for significant differences between treatments as described below.

### 2.3. RNA Sample Preparation

RNA from samples of pooled larvae stored at −80°C (one sample per vessel; four replicate vessels per treatment) was extracted using the NucleoSpin^®^ RNA Plus RNA isolation kit (Macherey-Nagel, Düren, Germany). RNA quantity and quality was checked with a NanoDrop^®^ ND-1000 Spectrophotometer (Thermo Fisher Scientific, Wilmington, DE, USA). Library preparation, sequencing, and initial analyses of RNA from the reciprocal transplant were performed by Novogene Corporation (UC Davis, Sacramento, CA, USA). RNA degradation and contamination were monitored on 1% agarose gels, RNA purity was checked using the NanoPhotometer spectrophotometer (IMPLEN, Westlake Village, CA, USA), and RNA integrity and quantitation were assessed using the RNA Nano 6000 Assay Kit of the Bioanalyzer 2100 system (Agilent Technologies, Santa Clara, CA, USA). For library preparation, 1 µg RNA per sample was used as input material and sequencing libraries were generated using NEBNext Ultra RNA Library Prep Kit for Illumina (NEB, Ipswich, MA, USA) with cDNA fragments of 150~200 bp in length preferentially selected for. Library fragments were purified with AMPure XP system (Beckman Coulter, Beverly, CA, USA). Then, 3 µL USER Enzyme (NEB, Ipswich, MA, USA) was used with size-selected, adaptor-ligated cDNA at 37 °C for 15 min followed by 5 min at 95 °C before PCR. Then, PCR was performed with Phusion High-Fidelity DNA polymerase, Universal PCR primers, and Index (X) Primer. Finally, PCR products were purified (AMPure XP system) and library quality was assessed on the Agilent Bioanalyzer 2100 system. The clustering of the index-coded samples was performed on a cBot Cluster Generation System using PE Cluster Kit cBot-HS (Illumina) before sequencing on an Illumina platform, where 125 bp/150 bp paired-end reads were generated.

### 2.4. RNAseq Data Processing

Novogene performed quality control, read mapping, quantification of gene expression level, differential expression analysis, Gene Ontology (GO) enrichment analysis of differentially expressed genes, and KEGG (Kyoto Encyclopedia of Genes and Genomes) pathway enrichment analysis of differentially expressed genes for RNAseq data. In the quality control step, clean reads were obtained by removing reads containing adapters, reads containing poly-N, and low quality reads from raw data. Q20, Q30, and GC content of the clean data were calculated. Cleaned data were used in downstream analyses. The reference genome and gene model annotation files were obtained from NCBI (NCBI Accession GCF_002022765.2) and the index of the reference genome was built using HISAT2 2.1.0. Paired-end clean reads were mapped to the reference using HISAT2 [43]. HTSeq v0.6.1 [44] was used to count the number of reads mapped to each gene and (fragments per kilobase of transcript per million mapped reads (FPKM) were calculated using the length of the gene and the number of reads mapping to that gene. Differential expression analysis was performed using the DESeq R package (1.18.0) [45], which uses a model based on the negative binomial distribution for determining differential expression. *p*-values were adjusted using the Benjamini and Hochberg approach for controlling the false discovery rate (FDR), and genes with a *p*-value < 0.05 were considered differentially expressed. Genes identified as differentially expressed were annotated using UniProtKB (UniProt Knowledgebase) and data were explored for promising genes/proteins related to processes that may be important for resilience (e.g., biomineralization, homeostasis, development) and compared to genes found in similar studies. Gene Ontology enrichment analysis was possible as the *C. virginica* transcriptome has previously been annotated. GO enrichment analysis of differentially expressed genes was implemented in the GOseq R package [46], in which gene length bias was corrected and GO terms with corrected *p*-values less than 0.05 were considered significantly enriched. KOBAS (KEGG Orthology-Based Annotation System) [47] software was used to test the statistical enrichment of differentially expressed genes in KEGG pathways.

### 2.5. Statistical Analysis

All statistical analyses were conducted using R statistical software v3.6.1. Assumptions of a normal distribution and homoscedasticity were confirmed using Shapiro–Wilk and Bartlett’s tests, respectively. Significant differences in viability between treatments were determined with a G-test of independence using William’s correction. Significant differences were concluded if *p* < 0.05 with post hoc pairwise G tests using Bonferroni correction. For growth analysis, data were compared using Student’s *t*-test (for normally distributed data) or a Kruskal–Wallis rank sum test followed by Mann–Whitney U test if the data did not conform to a normal distribution. In both cases, differences were considered significant at *p* < 0.05.

## 3. Results

### 3.1. Pre-Transplant

#### Viability and Growth

**Figure 1:**
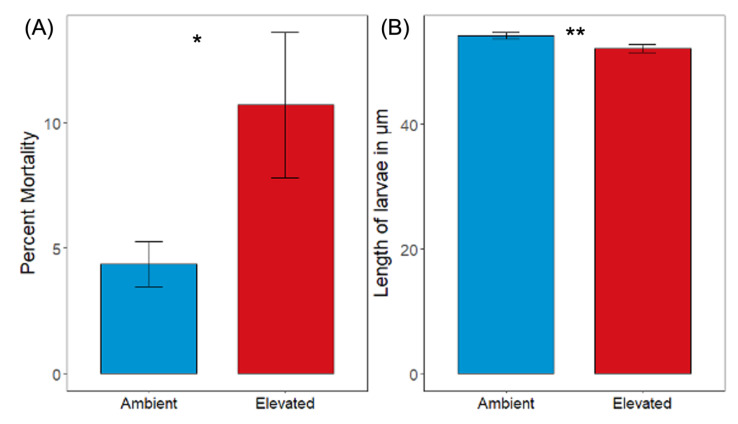
(**A**) Percent mortality for 7 day old oyster larvae before transplantation into the alternative *p*CO_2_ treatment (* denotes significant difference for (**A**), G-test of independence, *p* < 0.001; *n* = 4, with a minimum of 100 larvae per replicate). Error bars are the 95% confidence intervals. (**B**) Length in micrometers for 7 day old oyster larvae before transplantation into the alternative *p*CO_2_ treatment in the reciprocal transplant experiment (** denotes significant difference for (**B**), Mann–Whitney U test, *p* < 0.001; *n* = 4, with a minimum of 65 larvae per replicate). Error bars denote standard error of the mean.

Initial *p*CO_2_ had a significant effect on the mortality of larval oysters before transplantation into the alternative *p*CO_2_ treatment (G-test of independence, *G* = 47.459, *p* < 0.001; Figure 1a). Percent mortality was significantly higher for oysters under elevated *p*CO_2_ (10.70%) compared to oysters under ambient *p*CO_2_ (4.37%; Table S2).

Oysters under the ambient *p*CO_2_ treatment were significantly larger than those under the elevated *p*CO_2_ treatment (54.20 ± 0.498 and 52.11 ± 0.654 µm, respectively; Table S2; Mann–Whitney U test, *n* = 4, *p* < 0.001) (Figure 1b).

### 3.2. Post-Transplant

#### 3.2.1. Viability

**Figure 2:**
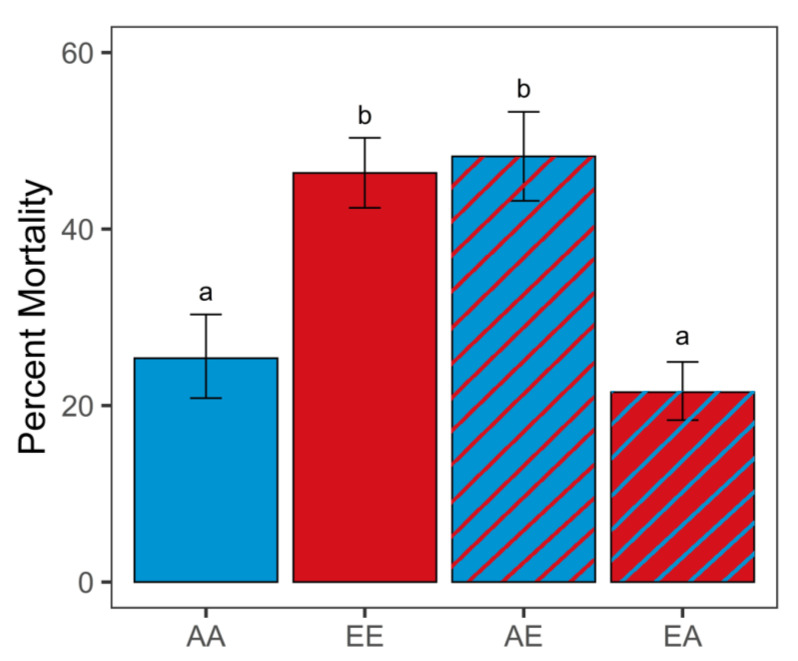
Percent mortality 1 week post-transplant (different letters a, b above error bars represent significant difference, G-test of independence, *p* < 0.001; *n* = 4 per treatment, with a minimum of 340 larvae per replicate). Error bars are the 95% confidence intervals. Sample code names: first letter indicates original *p*CO_2_ treatment (A: ambient, E: elevated); second letter indicates final *p*CO_2_ treatment (first and second letters are different for transplanted treatments).

Final post-transplantation *p*CO_2_ had a significant effect on the mortality of larval oysters (G-test of independence, *G* = 131.71, *p* < 0.001; Figure 2; Table S3). Percent mortality was significantly higher for non-transplanted elevated *p*CO_2_ oysters (EE, 46.35%) compared to ambient *p*CO_2_ oysters (AA, 25.36%; G-test of independence, *p* < 0.001). Oysters transplanted from elevated to ambient *p*CO_2_ conditions (EA) had significantly lower mortality (21.51%) compared to EE (G-test of independence, *p* < 0.001) and similar mortality compared to the control AA oysters (G-test of independence, *p* = 1). Oysters transplanted from ambient to elevated *p*CO_2_ conditions (AE) had significantly higher mortality (48.22%) compared to AA (G-test of independence, *p* < 0.001) and similar mortality compared to the EE oysters (G-test of independence, *p* = 1).

#### 3.2.2. Growth

**Figure 3:**
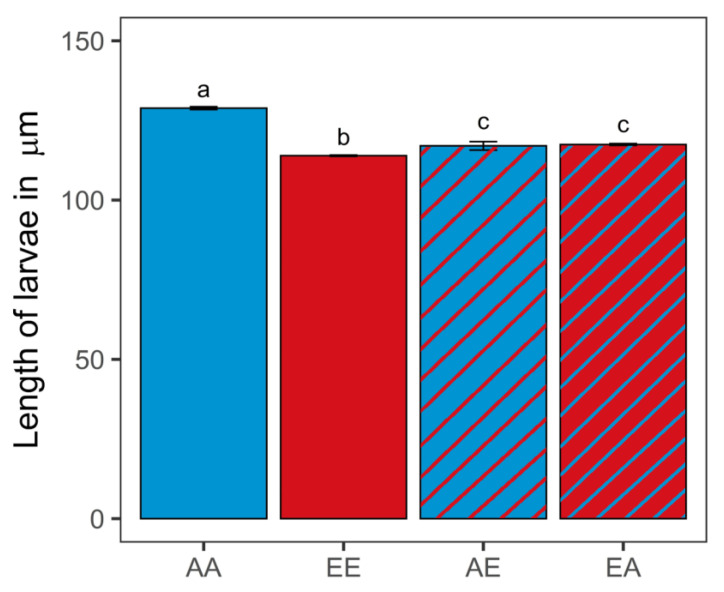
Length in micrometers for 15 day old oyster larvae transplanted (diagonal lines) and kept as controls (solid) (different letters a, b, c above error bars denote significant difference, Kruskal–Wallis rank sum test, *p* = 0.013; *n* = 4, with a minimum of 180 individuals per replicate). Error bars denote standard error of the mean. Sample code names: first letter indicates original *p*CO_2_ treatment (A: ambient, E: elevated); second letter indicates final *p*CO_2_ treatment (first and second letters are different for transplanted treatments).

Oysters in the control ambient *p*CO_2_ treatment (AA) were significantly larger than those in the non-transplanted elevated treatment (EE) (128.84 ± 0.442 and 113.91 ± 0.199 µm, respectively; Mann–Whitney U test, *n* = 4, *p* < 0.001) (Figure 3; Table S3). Oysters transplanted from the elevated treatment to the ambient *p*CO_2_ treatment (EA) were significantly larger (117.43 ± 0.271 µm) than EE oysters (Mann–Whitney U test, *n* = 4, *p* < 0.001). In contrast, oysters transplanted from the ambient to the elevated *p*CO_2_ treatment (AE) were significantly smaller (116.99 ± 1.340 µm) than the AA oysters (Mann–Whitney U test, *n* = 4, *p* < 0.001). There was no significant difference in size between oysters from the two transplanted treatments (AE vs. EA; Mann–Whitney U test, *n* = 4, *p* > 0.05).

### 3.3. Differential Gene Expression Analysis

**Figure 4:**
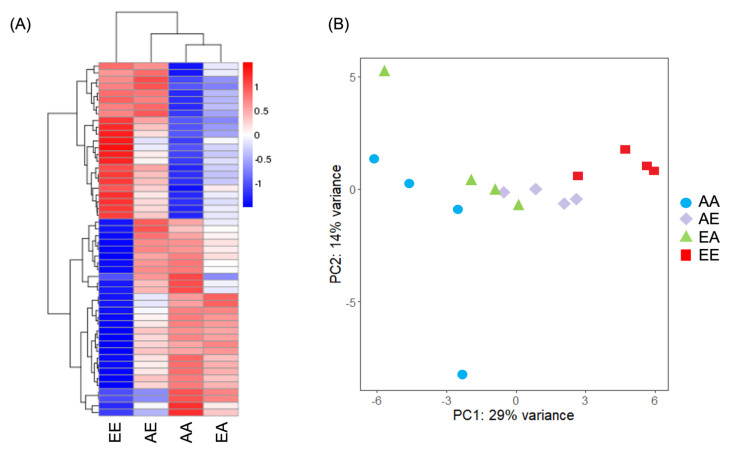
(**A**) Hierarchical cluster analysis of DEGs (*n* = 4 replicates per treatment coalesced into 1 column). Over- and under-expressed genes are represented in red and blue, respectively. Data are normalized by average expression in all samples. (**B**) Principal component analysis of the normalized RNASeq data. Sample code names: first letter signifies original *p*CO_2_ treatment (A: ambient, E: elevated); second letter signifies final *p*CO_2_ treatment (first and second letters are different for transplanted treatments).

**Figure 5:**
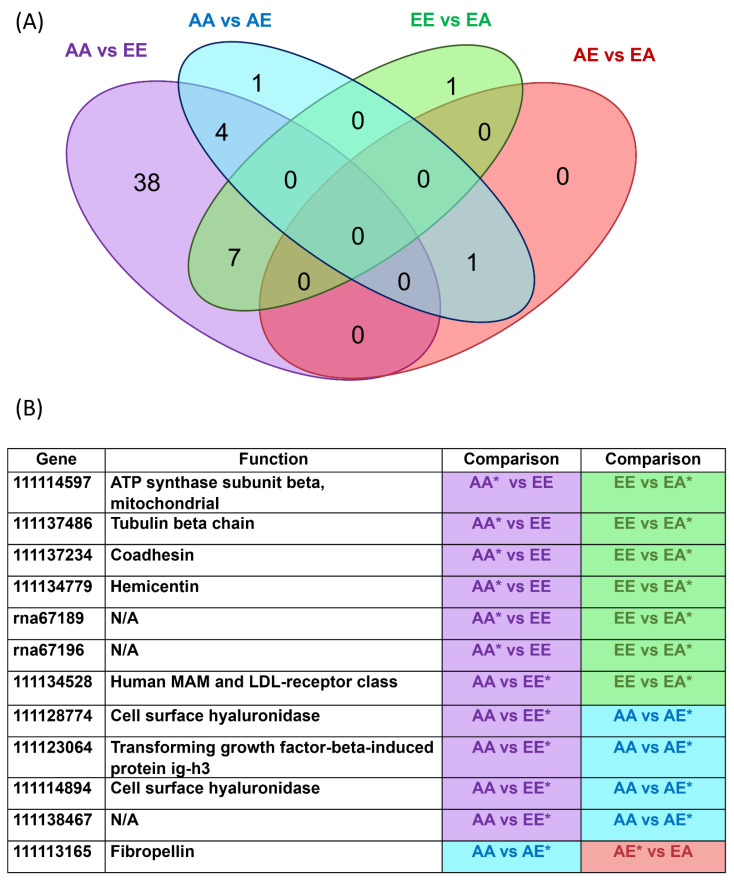
(**A**): Venn diagram showing the number of genes uniquely expressed within each group, with the overlapping regions showing the number of genes that are expressed in two or more groups. (**B**) DEGs in common/shared between comparisons; * indicates the group with higher expression for the gene. Purple: AA vs. EE; Blue: AA vs. AE; Green EE vs. EA; Red: AE vs. EA.

Pearson’s correlation coefficient comparisons of expression profiles demonstrated a moderate correlation (R^2^ > 0.314) for samples derived from the same treatment (Supplementary Figure S1). Using hierarchical clustering analysis (Figure 4a) and principal component analysis (Figure 4b), treatments were clustered based on the final *p*CO_2_ condition (i.e., non-transplanted ambient *p*CO_2_ samples (AA) and samples transplanted from elevated to ambient *p*CO_2_ (EA) were clustered most closely and non-transplanted elevated *p*CO_2_ samples (EE) and samples transplanted from ambient to elevated *p*CO_2_ (AE) were clustered together). The total number of differentially expressed genes (DEGs) between treatments ranged from 1 to 49 (Table 1, Table 2 and Table S4; Figure 5). The greatest number of DEGs was found to be between AA versus EE (the two stable conditions), with a total of 49 genes of which 21 were upregulated (i.e., relatively higher expression in EE) and 28 were downregulated (i.e., relatively lower expression in EE). The fewest number of DEGs was between AE and EA treatments, with a total of one gene which was upregulated in the AE treatment. AA versus AE and EE versus EA both had intermediate numbers of DEGs, with a total of six and eight DEGs, respectively. Of the 49 DEGs between AA and EE, 32 (65%) were annotated. In the comparison of AA versus AE, four of the six DEGs were annotated (66.7%). Of the eight DEGs between EE versus EA, five genes were annotated (62.5%). The single gene differentially expressed between AE and EA was also annotated.

Twelve genes up- or downregulated in a non-transplanted treatment were similarly expressed in larvae from a transplanted treatment (Figure 5). Nine of the eleven genes were annotated and had functions related to fibropellin, the mitochondrial ATP synthase β subunit, MAM and LDL-receptor class, tubulin β chain, coadhesin, hemicentin, and transforming growth factor-β-induced protein ig-h3 and two had hyaluronidase functions.

## 4. Discussion

The results of this study suggest phenotypic plasticity and acclimation contribute to the resilience to OA. The potential for recovery holds promise for the success of species management in the future. While pH is not expected to rebound rapidly in such a way that the recovery demonstrated here could occur in situ, the reciprocal transplant design provides insight into mechanisms for resilience. Our understanding of the ability for calcifying species exposed to OA conditions to rebound and recover normal physiological function is poor. The results of the reciprocal transplant experiment presented here suggest survival under OA conditions is due, at least in part, to phenotypic plasticity that enables larvae to survive under stressful conditions. The results also suggest that acclimation plays a role in governing resilience to elevated *p*CO_2_, which may enable the individual to tolerate the changing environmental conditions. Moreover, this conclusion sheds some light on the mechanisms larval oysters use to cope with cyclic periods of low pH, especially during summer periods with frequent and extreme fluctuations.

### 4.1. Effects of OA on C. virginica Larvae

Viability and growth in the non-transplanted treatments demonstrated higher mortality and smaller size for the oysters under elevated *p*CO_2_ versus those under ambient *p*CO_2_ (EE and AA, respectively), as expected based on previous studies [16]. Comparison between these two groups also showed the greatest number of DEGs. Taken together, these findings demonstrate the negative impacts of OA on *C. virginica* larvae and how they respond at the molecular level to alterations in carbonate chemistry. Larvae transplanted from ambient to elevated *p*CO_2_ conditions (AE) demonstrated significantly higher mortality and smaller size than oysters remaining under ambient *p*CO_2_ (AA). Their transcriptomic profiles were clustered based on final *p*CO_2_, so larvae at the end of the transplant terminating in OA conditions (i.e., EE and AE) generally had similar gene expression. While there was no difference in length between these larvae and larvae in the reverse transplant treatment (EA), they did have significantly greater mortality. A possible explanation for the higher mortality in AE as compared to EA is that young larvae are able to survive short-term exposure to high *p*CO_2_ before they become more susceptible to OA as they age and approach metamorphosis. Similar mortality in larvae between varied *p*CO_2_ conditions within the first few days of exposure has been found in other studies of Pacific and eastern oysters [16,48]. While we did see high mortality in the EE treatment compared to AA, an explanation for the greater mortality of AE vs. EA could be that the exposure of the larvae in the EA condition to OA conditions did not lead to immediate mortality and they were able to rebound in normal conditions, as opposed to older larvae that suffered higher mortality as they were moved from normal to acidified conditions.

### 4.2. Recovery of Larvae Returned to Normal Conditions

Many studies have demonstrated the impacts of OA on bivalves, but here we take it a step farther and look at what happens when oyster larvae are returned to normal conditions. Oyster larvae transplanted from elevated to ambient *p*CO_2_ conditions (EA) demonstrated significantly lower mortality and larger size than oysters remaining under elevated *p*CO_2_ (EE). The recovery of both viability and growth after transplantation in ambient conditions demonstrates the ability for larvae to rebound quickly (i.e., in one week) under normal conditions. The recovery is suggestive of the phenotypic plasticity of the eastern oyster, which has been demonstrated in other species with experiments of a similar design [27,29]. Recovery has been shown by Calosi et al. (2013) [6] in the polychaete species of *Amphiglena mediterranea*. Following transplantation of *A. mediterranea* from a natural CO_2_ vent system to ambient pH, metabolic rates increased back to levels equal to those of a population living outside the vent system after just five days. Calosi et al. (2013) [6] describe the results as indicative of phenotypic plasticity; however, the results were species-specific. For instance, *Platynereis dumerilii* (also a polychaete) individuals living within and outside of the vent system exhibited near equal metabolic rates, but transplantation to ambient CO_2_ conditions from elevated conditions resulted in an increase in rates above those of both non-transplanted populations. This suggests that *P. dumerilii* genetically adapted to living within a high CO_2_ vent environment, as the vent population was able to cope with elevated CO_2_ conditions. Here, *C. virginica* demonstrate a pattern more similar to *A. mediterranea* when transplanted to ambient *p*CO_2_ conditions from elevated conditions. This recovery would not have been observed had selection/adaptation been the mode of survival. If elevated *p*CO_2_ led only to the selection of individuals that tolerate low pH and resulted in a population that would be stressed under ambient conditions, oysters would not have rebound following transplantation to ambient *p*CO_2_. Instead, these results demonstrate phenotypic plasticity at a significant cost to normal physiological function (i.e., growth). The idea of costs or “trade-offs” between resilience traits is well-described in OA research and has been demonstrated in a number of species [13,49,50]. Often, survival under OA is described as the result of several trade-offs in physiological functions (i.e., growth, reproduction, immunity), but phenotypic plasticity can also be associated with trade-offs as it can result in the reallocation of energy budgets away from some functions [51]. However, if plasticity or acclimation becomes too “costly”, then selection may act to eliminate these genotypes in favor of more fit phenotypes and an adaptive mechanism for resilience would take over [52]. Bitter et al., 2019 [52] demonstrated substantial mortality in oyster larvae by day 26 of OA exposure and die-off of susceptible phenotypes, leaving a population of low-pH-tolerant oysters. The mechanism that confers resilience to OA in marine species is not only species-specific but may also change over time with chronic stress. In this study, a hatchery stock that was selectively bred for certain traits (such as disease resistance) was used. Durland et al., 2021 [53] demonstrated that larvae from aquacultured oyster stocks showed significantly less genetic changes than those derived from wild populations in response to OA, suggesting resilience to OA might also be population/stock-specific.

### 4.3. Transcriptomic Analyses

Analysis of gene expression levels revealed a shift in steady-state gene expression in oysters exposed to elevated *p*CO_2_. These results suggest acclimation as a mode for resilience to OA stress and demonstrate that *C. virginica* possesses the genetic repertoire needed for plasticity under altered environmental conditions. Overall, the trends demonstrate that larval transcriptomes respond to OA by downregulating genes involved in energy/metabolism and the cytoskeleton and genes dependent on calcium. Guided by prior studies [12,36,38,54,55,56] on related taxa, the differentially expressed genes (DEGs) between each treatment comparison were broken down into a small number of categories that highlighted the role they may play in resilience to OA. Here, only a select few genes are described, as these appear to be the most interesting genes for acclimation to OA. Most DEGs found in the reciprocal transplant experiment fit into one of four categories, many of which are related to biomineralization: (1) “energy/metabolic processes”, (2) components of the “extracellular matrix”, (3) the “cytoskeleton”, or (4) “immunity”.

#### 4.3.1. AA vs. EE

When comparing larvae from non-transplanted treatments, larvae predominantly downregulated genes under OA exposure (AA vs. EE). Multiple genes encoding proteins involved in cell differentiation and proliferation, including two cell surface hyaluronidases [57] and nucleolar GTP-binding protein, were upregulated in EE versus AA, which may have been an attempt to maintain growth and differentiation under elevated *p*CO_2_ stress. Two genes with proteins involved in ion exchange were also found to be upregulated in EE versus AA. Of these two genes, one, a 60 kDa neurofilament protein, was found to have a domain function in sodium/calcium exchange and the other, prestin, was found to encode a sulfate anion transporter. Evans et al. (2013) [58] and Todgham and Hofmann (2009) [24] found sodium/calcium exchangers to be upregulated in sea urchin under elevated *p*CO_2_ and suggested this may be in an effort to promote calcification [59]. Ramesh et al. (2019) [59] also identified the upregulation of sulfate-anion transporters in blue mussel larvae under OA and suggested this may be related to the need to uptake sulfate during shell biogenesis for the production of macromolecules in the organic matrix of the shell. With regard to immune-related genes, a gene encoding soma ferritin was found to be upregulated in EE versus AA. Soma ferritin serves in the oxidative stress response [60], and when reactive oxygen intermediate (ROI) production is high [61] under low pH, ferritin can mitigate the harmful effects on the system [62]. Alternatively, two genes associated with energy production (i.e., ATP synthesis) were downregulated in EE larvae when compared to AA larvae. A gene encoding an ATP synthase β subunit protein and a gene encoding an adenosylhomocysteinase A both encode proteins that function in aerobic and anaerobic energy metabolism and ATP synthase. A number of studies [63,64,65,66] found the downregulation of ATP synthase-related genes under elevated *p*CO_2_, which suggests OA alters metabolism in calcifiers. Contrarily, ATP synthase was significantly upregulated in low pH conditions in Crassostrea *gigas* oyster larvae, suggesting larvae experiencing OA conditions also had altered metabolism, but in this case they increased their energy production [67].

#### 4.3.2. Expression Levels by Final pCO_2_ Treatment

Interestingly, all genes identified as DEGs for the three other comparisons (AA versus AE, EE versus EA, and AE versus EA) were represented in the AA versus EE comparison, with the exception of one gene which encoded fibropellin (which was identified as a DEG in AA versus AE and also AE versus EA). This led to an interesting pattern in the data: 12 genes that were either up or downregulated in a non-transplanted treatment were similarly expressed in larvae from a transplanted treatment. To this extent, the differential regulation of some genes in the AE transplanted treatment converged with the regulation of the EE treatment with regard to the baseline expression in AA; in other words, the expression level of a select few genes was the same for both treatments terminating in elevated *p*CO_2_ (EE and AE) with regard to the baseline expression in AA. The same was true for the regulation of some genes in the EE treatment with regard to the baseline expression in both AA and EA; in other words, the expression of some genes was the same for both treatments terminating in ambient *p*CO_2_ (AA and EA) with regard to the expression in EE. Thus, transplanted larvae had expression profiles that resembled the patterns of expression demonstrated by non-transplanted larvae within one week of transplantation. This is supported by the cluster analysis, in which treatments were clustered most closely based on final *p*CO_2_ treatment. In a study performed by [68], a similar pattern of shared expression was found for transplanted versus native guppies, though the study looked at patterns of expression in the absence of predators and not environmental stress. The study transplanted guppies adapted to living in the presence of predatory cichlids to a predator-free habitat and compared gene expression with a guppy population that evolved in the absence of predators. The authors found 135 transcripts that exhibited up- or downregulation in the same direction as the native cichlid-free population. The authors concluded that this was demonstrative of rapid changes in plasticity. Similarly, [69] found convergence of gene expression profiles for transplants and natives in a reciprocal transplant experiment in which three-spined stickleback (*Gasterosteus aculeatus*) were transplanted to foreign environments. The authors also proposed that this convergence suggested expression plasticity, but they noted that fish from different habitats still differed in survival rates and infection rates after transplant.

##### AA vs. EE and AA vs. AE

Of the genes held in common in comparisons between AA and EE and AA and AE, three genes, encoding two cell surface hyaluronidases and the transforming growth factor-β-induced protein ig-h3, were upregulated in EE and AE with respect to AA. Cell surface hyaluronidase is important for maintaining protein homeostasis and surviving protein misfolding stress [70]. Improved cellular stress resistance from cell-surface hyaluronidase-like proteins was suggested to be a cellular stress response mechanism, allowing tolerance to environmental perturbations in Sydney rock oyster [71]. In a study of highly expressed genes of *C. gigas* larvae, hyaluronidase-related sequences were suggested to be important to the extracellular matrix, specifically remodeling of the extracellular matrix [72]. Production of hyaluronan increases in proliferating cells and plays an important role in development and differentiation. Interestingly, TMEM2 hyaluronidase (now CEIP2) is dependent on calcium as a cofactor [57]. The upregulation of hyaluronidase may be the result of an increase in hyaluronan in an attempt to mitigate the effects on development under OA stress. Studies have documented delayed development in calcifiers [10], but the upregulation of this gene may be an attempt to achieve homeostasis in larvae under acidic conditions. The second of the two types of upregulated genes was a gene that encodes transforming growth factor-β-induced protein ig-h3, which is a fascilin protein and a member of a family of cell adhesion molecules. Fascilin shares features with immunoglobulins, cadherins, integrins, and selectins. The function of fascilin in oysters is unknown; however, the purpose of the upregulation of the gene encoding the protein may be hypothesized based on the function of other cell adhesion molecules. Immunoglobulins, integrins, and selectins all play important functions in the cell, but, under OA stress, cadherins may be the most interesting molecules to examine. Cadherins are cell adhesion molecules dependent on calcium and play a role in morphogenesis and homeostasis. This is particularly interesting given the upregulation of hyaluronidase, another calcium-dependent molecule with a function in development and differentiation. Together, these genes may be important in sustaining the development of larvae even when development is disrupted or slowed under OA.

##### EE vs. AA and EE vs. EA

Of the genes held in common in the comparisons between EE and AA and EE and EA, four genes, encoding the mitochondrial ATP synthase β subunit, tubulin β chain, coadhesin, and hemicentin, were downregulated in EE with respect to both AA and EA. Overall, downregulated genes may be interpreted as “costs” under OA. The gene encoding the mitochondrial ATP synthase β subunit is important for aerobic energy production through its functioning in the synthesis of ATP, the molecule responsible for storing and transporting chemical energy in cells [73]. As suggested in [64], in which larval *C. gigas* demonstrated lower expression of ATP synthase, decreased expression suggests altered metabolism under acidification. A gene encoding tubulin β chain was also downregulated under EE with respect to AA and EA. Tubulin is a major constituent of microtubules and is associated with the cytoskeleton [74]. In Müller et al. (2018) [74], *Crassostrea brasiliana* exposed to a diesel fuel water-accommodated fraction differentially regulated α and β tubulin. This differential expression was cited to be the result of oxidative stress, which could explain the downregulation in larvae here. Coadhesin has been found to be a component of the skeletal organic matrix of the staghorn coral *Acropora millepora* [75], and thus the downregulation of this may be related to the smaller size demonstrated in the physiological assay. Similarly, the fourth downregulated gene encodes hemicentin, which was also found in *A. millepora* skeletal organic matrix [75] and was downregulated in the coral *A. gemmifera* after exposure to high CO_2_ conditions [8]. Additionally, one of the GO-molecular functions associated with hemicentin is calcium-ion binding, which may provide even further information as to why the specific gene is downregulated under OA stress.

Though hemicentin may have calcium dependency, interestingly, most of the calcium-dependent enzymes identified as DEGs were found to be upregulated under elevated *p*CO_2_ conditions. The CEIP2 gene encoding a cell surface hyaluronidase and the gene encoding the transforming growth factor β-induced ig-h3 were identified as calcium-dependent and were both upregulated in both EE and AE versus AA. Additionally, the EGF1 gene, which encodes the protein fibropellin-1, was upregulated in AE when compared to both the AA and EA conditions. Fibropellin-1 is characterized by a calcium-binding EGF-like domain, which results in a protein dependent on calcium for proper biological function [76]. These results suggest not only that larval oysters are responding to their present environment by means of a plastic response (as is the case with the expression of CEIP2 in treatments terminating in elevated *p*CO_2_), but also that one of the key systems important in tolerating the stress of OA is dependent on the presence of calcium.

#### 4.3.3. GO Enrichment

Currently, GO categories are not optimized for non-model marine invertebrates, which results in gene groups that seemingly have no relation to oyster homeostasis or development. For this reason, it can be difficult to relate the GO categories identified as significant to a specific function that confers resilience under acidification stress. The three significant GO terms enriched in AA were related to arabinosyltransferase activity, actinobacterium-type cell wall biogenesis, and peptidoglycan-based cell wall biogenesis (Table S5). The significance of these terms remains unclear. Similar to the GO analysis, KEGG is not optimized for non-model marine invertebrates, and so many enrichment pathways have seemingly unrelated functions; however, some investigation of the pathways is still possible (Table S6). Between AA and EE, the oxidative phosphorylation pathway was downregulated in EE oysters and was represented by two DEGs encoding ATP synthase. The downregulation of this pathway is likely related to the altered metabolism suggested for calcifiers under OA stress [63,64,65].

#### 4.3.4. Molecular Features Associated with Resilience to OA

Differences in gene expression may provide some information as to the genes affected under environmental stress; however, the degree to which stressed and control populations differ does not always indicate tolerance versus susceptibility [77]. In some species, the more tolerant population exhibits greater differential expression between stressed and control treatments (i.e., the seagrass *Zostera marina*; [78] spiny damselfish *Acanthochromis polyacanthus* [79]) whereas, in other species, it is the more sensitive population that exhibits greater differential expression when stressed (i.e., coral [80]). Therefore, adaptive pressure can act in different ways on different species to result in changes in the ability of a species/population to acclimate via changes in gene expression. To this degree, caution must be taken when discussing the results of gene expression studies, as interpreting the results can be complicated. The differences between larval transcriptomes could reflect higher susceptibility in the younger life stage, as this is supported by the results of the physiological assay (decreased viability and growth) demonstrated earlier. In addition, this conclusion is supported by the types of genes/proteins regulated; in larvae, changes in expression are associated with alteration of basic functions, such as ATP synthase, fascilin proteins, ion transporters, and immune-related proteins; thus, larvae are deemed susceptible overall. Despite the complexity in interpreting DEGs, transcriptional plasticity can be an important component underlying resilience and acclimation to stress. Ultimately the modification of plastic responses can lead to adaptation to climate change if the stress of OA leads to directional selection on expression level of DEGs. It can, therefore, be difficult to parse the roles of acclimation and adaptation when the modes of resilience can select for one another.

Transcriptomic analyses support the results demonstrated in the physiological assays and previous studies [7,8,16], which suggest larvae are susceptible to the effects of OA and the demonstrated resilience in surviving larvae is due, in part, to changes in gene expression. Studies investigating DEGs between two populations under environmental stress can often differ in their results due to biological variability, differences in experimental methods (e.g., age of individuals, source population, and time of sample collection [31]), and the sensitivity of NGS methods, but many genes identified here, including ATP synthase, sodium/calcium exchangers, and heat shock proteins, were also identified in other OA studies employing RNAseq [24,54,58,62,63,64]. These genes and the proteins they encode may be considered the most promising genes for resilience under OA stress and represent targets for the next step in understanding mechanisms for resilience.

A common question surrounding OA research asks about the rate with which these changes (i.e., phenotypic plasticity or acclimation) are expected to take place as compared to the pace of climate change. The present study begins to answer this question. If larval oysters can acclimate to a new pH regime within a period of approximately one week, then there may be hope for their success under future ocean conditions. However, though this experiment demonstrated oysters exhibit phenotypic plasticity under OA stress, this leads to further questions regarding whether other, less energetically costly adaptive mechanisms are possible. The ability to acclimate does not mutually exclude an adaptive mechanism and the physiological assays presented here do not probe all of the potential molecular processes for acclimation. As acclimation potential has a genetic component [77], investigating the genome of *C. virginica* under OA stress for changes in allele frequency may be crucial to comprehensively describe oyster resilience to OA. Identifying the presence of selection at the genome level (i.e., single-nucleotide polymorphisms (SNPs)) can serve to enhance and complement our understanding of the molecular features associated with resilience to OA in the larval and juvenile eastern oyster. Although phenotypic plasticity was observed in this study, it can have other implications and is difficult to interpret [81]. While we hypothesized that the regulation of some of the identified genes provides an increased tolerance to acidification, further research needs to be conducted to validate these findings, such as utilizing gene silencing/editing approaches to determine if identified genes are effectively vital for resilience to OA.

## 5. Conclusions

This study combined physiological assays with next-generation sequencing to assess the potential for recovery from and acclimation to OA in *C. virginica* larvae and identified molecular mechanisms associated with resilience. Oyster larvae that were transplanted from OA conditions to ambient conditions had significantly lower mortality and larger size than oysters remaining in OA conditions. This demonstrates that larvae were able to recover and rebound quickly when returned to ambient conditions. The recovery is suggestive of the phenotypic plasticity of the eastern oyster. In parallel, larvae reared under ambient conditions and transplanted to OA conditions showed a convergence in phenotypic profiles with those continuously held under OA. Transcriptomic analysis supported these findings, as DEG profiles of larvae post-transplant terminating in the same final *p*CO_2_ treatment converged, further supporting the idea that acclimation is a mechanism of resilience to OA. Analysis of gene expression levels demonstrated that larval transcriptomes respond to OA by downregulating genes involved in energy/metabolism and the cytoskeleton and genes dependent on calcium. Altogether, these findings contribute to a better understanding of the mechanisms involved in oyster resilience to OA and pave the way to the development of strategies to optimize the production of oyster stocks for aquaculture or restoration needs.

## Figures and Tables

**Table 1:** DEGs of non-transplanted (AA vs. EE) oyster larvae. Positive and negative Log2 fold changes indicate genes over- or under-expressed in EE as compared to AA, respectively.

Gene ID	Gene Description	Log2FC	padj
111100330	*mucin-5AC-like*	1.25	0.00
111100642	*uncharacterized LOC111100642*	1.94	0.00
111101544	*mucin-2-like*	1.43	0.01
111103490	*elongation factor 2-like*	2.16	0.05
111114894	*cell surface hyaluronidase-like*	1.93	0.00
111117016	*eppin-like*	2.00	0.00
111117698	*uncharacterized LOC111117698*	3.32	0.00
111120277	*plasminogen-like*	1.10	0.00
111122709	*serine protease inhibitor Cvsi-2-like*	1.82	0.01
111123064	*transforming growth factor-β-induced protein ig-h3-like*	2.10	0.00
111125413	*nucleolar GTP-binding protein 1-like*	1.22	0.00
111126276	*uncharacterized LOC111126276*	1.71	0.00
111126433	*70 kDa neurofilament protein-like*	1.62	0.02
111127373	*uncharacterized LOC111127373*	1.33	0.00
111128055	*countin-1-like*	1.94	0.00
111128774	*cell surface hyaluronidase-like*	2.39	0.01
111134162	*sulfate anion transporter 1-like*	2.11	0.02
111134528	*meprin A subunit β-like*	1.49	0.00
111138040	*soma ferritin-like*	1.02	0.01
111138380	*uncharacterized LOC111138380*	2.57	0.00
111138467	*uncharacterized LOC111138467*	0.97	0.01
111101484	*glycine-rich cell wall structural protein 2-like*	−2.21	0.00
111106582	*survival motor neuron protein-like*	−6.53	0.00
111109677	*high mobility group protein B3-like*	−1.80	0.02
111114597	*ATP synthase subunit β, mitochondrial*	−1.60	0.00
111118395	*uncharacterized LOC111118395*	NA	0.01
111120247	*ABC transporter F family member 4-like*	−4.27	0.05
111123230	*coadhesin-like*	−6.00	0.00
111125329	*senecionine N-oxygenase-like*	−2.70	0.02
111125850	*carnosine synthase 1-like*	−1.27	0.03
111125973	*actin, adductor muscle*	−1.45	0.05
111126272	*fibrocystin-L-like*	−2.61	0.00
111127289	*heat shock cognate 71 kDa protein*	−0.87	0.03
111127865	*uncharacterized LOC111127865*	−3.20	0.00
111128647	*chymotrypsin-like elastase family member 2A*	−3.23	0.01
111131442	*uncharacterized LOC111131442*	−1.85	0.00
111132959	*uncharacterized LOC111132959*	−2.37	0.02
111134775	*SCO-spondin-like*	−1.33	0.00
111134777	*coadhesin-like*	−4.42	0.02
111134779	*A disintegrin and metalloproteinase with thrombospondin motifs adt-1-like*	−2.09	0.00
111134780	*SCO-spondin-like*	−2.43	0.00
111134925	*myosin regulatory light chain A, smooth adductor muscle-like*	−1.16	0.05
111135192	*adenosylhomocysteinase A-like*	−1.39	0.00
111135859	*Y-box factor homolog*	−1.15	0.01
111137234	*SCO-spondin-like*	−2.51	0.00
111137486	*tubulin β chain*	−0.94	0.00
111138187	*ATP synthase lipid-binding protein, mitochondrial-like*	−1.55	0.01

**Table 2:** DEGs detected among transplanted (AA vs. AE; EE vs. EA; AE vs. EA) oyster larvae. Positive Log2 fold changes indicate genes over-expressed in the second group within each pair.

Gene ID	Gene Description	Log2FC	padj
AA vs. AE			
111113165	*fibropellin-1-like*	1.75	0.00
111114894	*cell surface hyaluronidase-like*	2.04	0.00
111121938	*uncharacterized LOC111121938*	1.62	0.02
111123064	*transforming growth factor-β-induced protein ig-h3-like*	2.00	0.02
111128774	*cell surface hyaluronidase-like*	2.90	0.00
111138467	*uncharacterized LOC111138467*	1.35	0.01
EE vs. EA			
111114597	*ATP synthase subunit β, mitochondrial*	1.35	0.02
111134779	*A disintegrin and metalloproteinase with thrombospondin motifs adt-1-like*	1.20	0.01
111137234	*SCO-spondin-like*	2.01	0.00
111137486	*tubulin β chain*	0.67	0.01
111134528	*meprin A subunit β-like*	−0.90	0.01
AE vs. EA			
111113165	*fibropellin-1-like*	−1.46	0.01

## Data Availability

The original contributions presented in the study are publicly available. Transcriptomics data can be found at https://www.ncbi.nlm.nih.gov/sra (accessed on 25 June 2022) under the accession number SAMN29371853.

## Supplementary Materials

The following supporting information can be downloaded at: https://www.mdpi.com/article/10.3390/genes13091529/s1, Figure S1: Pearson’s correlation coefficient matrix for all samples included in the DEG analysis; Table S1: Seawater chemistry values ± SD; Table S2: Average size and mortality of larvae in each tank at time 0 (before reciprocal transplant); Table S3: Average size and mortality of larvae in each tank after reciprocal transplant; Table S4: DEGs for the four comparisons: AA vs. EE, AA vs. AE, EE vs. EA, and AE vs. EA; Table S5: Over-represented gene ontologies; Table S6: Enriched pathways in the reciprocal transplant experiment.
