# Supplementary material for: A Transcriptomic Analysis of Phenotypic Plasticity in Crassostrea virginica Larvae under Experimental Acidification
Source: Genes (Basel). 2022 Aug 25;13(9):1529. doi: 10.3390/genes13091529 (PMC9498863; doi:10.3390/genes13091529)
Supplement: Supplementary file 1 [file genes-13-01529-s001.zip › genes-1813483-supplementary.pdf]

Supplementary Information

Dissolved Inorganic Carbon

Details of water chemistry conditions are provided in Table S1. Carbonate chemistry matched the target pCO_2 for the ambient and elevated treatments. pH levels in the treatment were approximately 8.1 and 7.5 for the ambient and elevated treatments, respectively. In the elevated pCO_2 condition aragonite was undersaturated.

Table S1. Seawater chemistry values ± SD. * denotes values calculated with *seacarb.*

Treatment	pH	pCO_2 (ppm)*	$\Omega_{aragonite}$*	$\Omega_{calcite}$*	DIC*	CO_3 (µmol/kg)*	ALK(µeq/kg)*
Ambient	8.14±0.08	322.57±78.74	3.36±0.54	5.20±0.83	1981.30±35.41	209.07±33.58	2268.11±67.95
Elevated	7.49±0.09	1600.23±346.23	0.83±0.15	1.28±0.24	2060.09±44.85	51.61±9.49	2084.47±46.61

Results of mortality and growth of larvae in each replicate tank

Table S2. Average size and mortality of larvae in each tank at Time 0 (before reciprocal transplant)

Treatment	Tank	Size T0	Mortality (%) T0
**Ambient**	1	54.96	5.69
**Ambient**	2	55.13	3.99
**Ambient**	3	53.15	4.17
**Ambient**	4	53.55	3.61
**Elevated**	1	50.62	14.21
**Elevated**	2	53.39	7.00
**Elevated**	3	53.02	10.53
**Elevated**	4	51.42	10.03

Table S3. Average size and mortality of larvae in each tank after reciprocal transplant

Treatment	Tank	Size	Mortality (%)
AA	1	129.54	30.71
AA	2	127.59	23.73
AA	3	128.86	31.51
AA	4	129.38	13.10
AE	1	113.08	45.00
AE	2	119.18	31.63
AE	3	117.82	65.74
AE	4	117.87	50.00
EE	1	113.64	52.38
EE	2	114.19	32.50
EE	3	113.50	34.78
EE	4	114.30	59.32
EA	1	117.63	28.81
EA	2	116.67	19.13
EA	3	117.48	18.90
EA	4	117.93	21.03

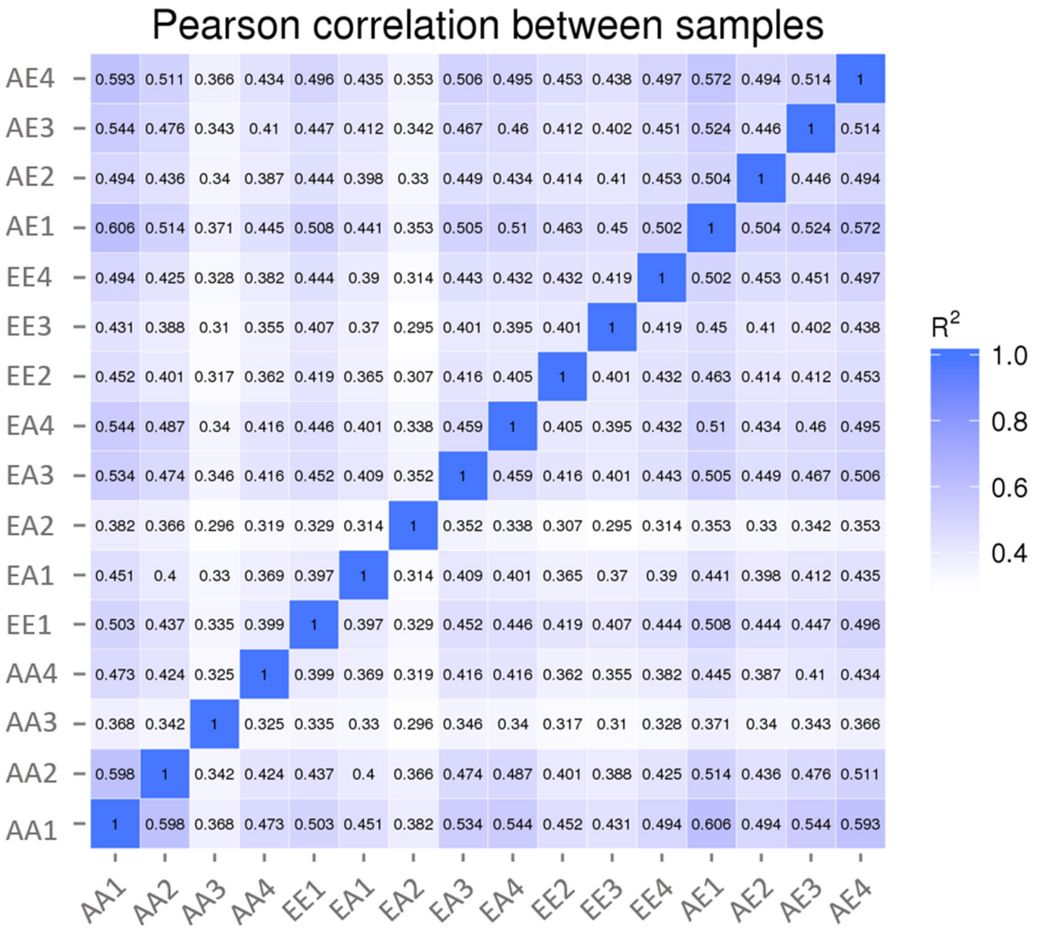

Figure S1. Pearson's correlation coefficient matrix for all samples included in the DEG analysis. Sample code names: First letter signifies original pCO_2 treatment (A: Ambient, E: Elevated); Second letter signifies final pCO_2 treatment (first and second letters are different for transplanted treatments); Number: replicate number.

Table S4. DEGs for the 4 comparisons: AA vs EE, AA vs AE, EE vs EA, and AE vs EA. Positive Log2 fold changes indicate genes over-expressed in the second group within each pair.

**AA versus EE**						
Gene_id	readcount_AA	readcount_EE	log2FoldChange	pval	padj	Blast swiss prot
Upregulated						
111100330	88.69611177	210.8158723	1.249	6.95E-06	0.00458	-//-
111100642	19.91430259	76.3138497	1.9381	2.38E-06	0.002075	-//-
111101544	51.68508115	139.7061414	1.4346	2.21E-05	0.011235	-//-
111103490	12.76684569	56.88007324	2.1555	0.00012	0.045398	sp|Q90705|EF2_CHICK Elongation factor 2 OS=Gallus gallus OX=9031 GN=EEF2 PE=1 SV=3//0
111114894	44.53737794	169.3873295	1.9272	4.66E-10	1.18E-06	sp|Q5FWI3|TMEM2_MOUSE Cell surface hyaluronidase OS=Mus musculus OX=10090 GN=Tmem2 PE=1 SV=1//4.31771e-106
111117016	16.09444219	64.52559379	2.0033	1.65E-06	0.001548	sp|P0DN10|VKT6_ANEVI U-actitoxin-Avd3i OS=Anemonia viridis OX=51769 PE=3 SV=1//9.87077e-17
111117698	3.725498611	37.1461754	3.3177	3.24E-06	0.002507	-//-
111120277	77.23102117	165.0218308	1.0954	6.08E-06	0.004159	sp|Q7Z410|TMPS9_HUMAN Transmembrane protease serine 9 OS=Homo sapiens OX=9606 GN=TMPRSS9 PE=1 SV=2//2.09781e-73
111122709	36.47136117	128.7121621	1.8193	1.34E-05	0.007442	-//-
111123064	20.07882573	85.94842481	2.0978	6.66E-07	0.000849	sp|P55906|BGH3_BOVIN Transforming growth factor-beta-induced protein ig-h3 OS=Bos taurus OX=9913 GN=TGFBI PE=1 SV=2//1.51711e-13
111125413	160.001551	371.7078839	1.2161	1.90E-07	0.000338	sp|Q9BZE4|NOG1_HUMAN Nucleolar GTP-binding protein 1 OS=Homo

						sapiens OX=9606 GN=GTPBP4 PE=1 SV=3//0
111126276	47.55222239	155.6331108	1.7106	1.57E-06	0.001548	-//-
111126433	29.72254076	91.0518816	1.6151	5.53E-05	0.022877	sp|Q01240|NF60_DORPE 60 kDa neurofilament protein OS=Doryteuthis pealeii OX=1051067 PE=2 SV=1//3.69139e-167
111127373	143.2049468	360.539014	1.3321	3.09E-06	0.002498	-//-
111128055	22.51479553	86.37491729	1.9397	2.83E-07	0.000457	sp|Q86IV5|CTNA_DICDI Countin-1 OS=Dictyostelium discoideum OX=44689 GN=ctnA PE=1 SV=1//1.21276e-36
111128774	9.150987731	47.98239301	2.3905	2.79E-05	0.013773	sp|Q9UHN6|TMEM2_HUMAN Cell surface hyaluronidase OS=Homo sapiens OX=9606 GN=TMEM2 PE=1 SV=1//8.47923e-104
111134162	12.96886282	55.90368249	2.1079	5.28E-05	0.022351	sp|Q9JKQ2|S26A5_MERUN Prestin OS=Meriones unguiculatus OX=10047 GN=SLC26A5 PE=1 SV=1//8.89433e-108
111134528	194.5807605	547.5841334	1.4927	6.03E-12	2.65E-08	sp|Q5VYJ5|MALR1_HUMAN MAM and LDL-receptor class A domain-containing protein 1 OS=Homo sapiens OX=9606 GN=MALRD1 PE=1 SV=4//3.73958e-12
111138040	225.3140205	456.8195304	1.0197	1.41E-05	0.007575	sp|P42577|FRIS_LYMST Soma ferritin OS=Lymnaea stagnalis OX=6523 PE=2 SV=2//7.68733e-92
111138380	10.41983719	61.85835523	2.5696	5.13E-07	0.00076	-//-
111138467	320.9177089	627.3314496	0.96702	8.17E-06	0.005188	-//-
Downregulated						

111101484	178.3572098	38.51857784	-2.2111	7.45E-12	2.65E-08	-//-
111106582	17.2093693	0.186750842	-6.5259	5.39E-06	0.003864	-//-
111109677	59.03435919	16.94737493	-1.8005	4.34E-05	0.020316	sp|P07746|HMGT_ONCMY High mobility group-T protein OS=Oncorhynchus mykiss OX=8022 PE=2 SV=2//1.16947e-18
111114597	184.1920586	60.70997762	-1.6012	6.69E-07	0.000849	sp|Q05825|ATPB_DROME ATP synthase subunit beta, mitochondrial OS=Drosophila melanogaster OX=7227 GN=ATPsynbeta PE=1 SV=3//0
111118395	14.30560391	0	Inf	1.12E-05	0.006428	-//-
111120247	16.08723956	0.831811824	-4.2735	0.000123	0.045398	-//-
111123230	21.27666316	0.332724729	-5.9988	7.43E-07	0.00088	sp|Q96RW7|HMCN1_HUMAN Hemicentin-1 OS=Homo sapiens OX=9606 GN=HMCN1 PE=1 SV=2//1.74072e-46
111125329	32.98025412	5.067092044	-2.7024	5.20E-05	0.022351	sp|Q8MP06|SNO1_TYRJA Senecionine N-oxygenase OS=Tyria jacobaeae OX=179666 GN=sno1 PE=1 SV=1//6.40554e-61
111125850	240.5462489	100.0921112	-1.265	8.77E-05	0.034654	sp|A5YM72|CRNS1_HUMAN Carnosine synthase 1 OS=Homo sapiens OX=9606 GN=CARNS1 PE=1 SV=3//2.48936e-180
111125973	520.6706868	190.2687231	-1.4523	0.000121	0.045398	sp|Q26065|ACT_PLAMG Actin, adductor muscle OS=Placopecten magellanicus OX=6577 PE=2 SV=1//0
111126272	87.3477924	14.2946033	-2.6113	1.08E-09	2.40E-06	sp|Q80ZA4|PKHL1_MOUSE Fibrocystin-L OS=Mus musculus OX=10090 GN=Pkhd1l1 PE=1 SV=1//0

111127289	459.8336847	251.4168017	-0.87103	8.72E-05	0.034654	sp|Q9U639|HSP7D_MANSE Heat shock 70 kDa protein cognate 4 OS=Manduca sexta OX=7130 PE=2 SV=1//0
111127865	105.4211755	11.50627458	-3.1957	1.03E-12	6.13E-09	-//-
111128647	34.19475994	3.637505291	-3.2328	8.61E-06	0.005278	sp|P00773|CELA1_RAT Chymotrypsin-like elastase family member 1 OS=Rattus norvegicus OX=10116 GN=Cela1 PE=1 SV=1//2.14068e-48
111131442	121.2944165	33.72761368	-1.8465	1.02E-06	0.001138	sp|B3EWZ3|CADN_ACRMI Coadhesin (Fragment) OS=Acropora millepora OX=45264 PE=1 SV=1//2.18435e-06
111132959	45.99994804	8.927179544	-2.3654	4.14E-05	0.019894	-//-
111134775	342.373583	135.7700571	-1.3344	1.33E-07	0.000262	sp|Q96RW7|HMCN1_HUMAN Hemicentin-1 OS=Homo sapiens OX=9606 GN=HMCN1 PE=1 SV=2//1.10244e-47
111134777	19.97423914	0.933754208	-4.419	4.98E-05	0.022351	sp|D3YXG0|HMCN1_MOUSE Hemicentin-1 OS=Mus musculus OX=10090 GN=Hmcn1 PE=1 SV=1//1.87326e-26
111134779	385.5610708	90.73851437	-2.0872	2.43E-14	2.16E-10	sp|Q96RW7|HMCN1_HUMAN Hemicentin-1 OS=Homo sapiens OX=9606 GN=HMCN1 PE=1 SV=2//1.53322e-44
111134780	136.7151343	25.45445569	-2.4252	2.38E-10	7.04E-07	sp|B3EWZ3|CADN_ACRMI Coadhesin (Fragment) OS=Acropora millepora OX=45264 PE=1 SV=1//4.35e-19
111134925	153.2693434	68.6577348	-1.1586	0.000138	0.049919	sp|P04113|MLRA_MIZYE Myosin regulatory light chain A, smooth adductor muscle OS=Mizuhopecten

						yessoensis OX=6573 PE=1 SV=1//1.63683e-74
111135192	245.9214829	93.6219809	-1.3933	5.43E-06	0.003864	sp|P51893|SAHHA_XENLA Adenosylhomocysteinase A OS=Xenopus laevis OX=8355 GN=ahcy-a PE=2 SV=1//0
111135859	315.4422657	142.5180152	-1.1462	1.05E-05	0.006241	sp|P41824|YBOXH_APLCA Y-box factor homolog OS=Aplysia californica OX=6500 PE=2 SV=1//3.10543e-44
111137234	253.0849586	44.52282664	-2.507	9.21E-15	1.64E-10	sp|B3EWZ3|CADN_ACRMI Coadhesin (Fragment) OS=Acropora millepora OX=45264 PE=1 SV=1//3.0659e-27
111137486	2630.939326	1372.765336	-0.93849	2.45E-06	0.002075	sp|P11833|TBB_PARLI Tubulin beta chain OS=Paracentrotus lividus OX=7656 PE=2 SV=1//0
111138187	106.3470696	36.28579149	-1.5513	1.60E-05	0.00837	sp|Q5RAP9|AT5G2_PONAB ATP synthase F(0) complex subunit C2, mitochondrial OS=Pongo abelii OX=9601 GN=ATP5MC2 PE=2 SV=1//6.41103e-21
**AA versus AE**						
Gene_id	readcount_AA	readcount_AE	log2FoldChange	pval	padj	Blast swiss prot
Upregulated						
111113165	110.2138444	371.9782772	1.7549	8.96E-10	1.69E-05	sp|P10079|FBP1_STRPU Fibropellin-1 OS=Strongylocentrotus purpuratus OX=7668 GN=EGF1 PE=1 SV=2//3.1654e-07
111114894	58.43435151	240.5317121	2.0413	1.40E-08	0.000131	sp|Q5FWI3|TMEM2_MOUSE Cell surface hyaluronidase OS=Mus

						musculus OX=10090 GN=Tmem2 PE=1 SV=1//4.31771e-106
111121938	44.8220669	137.6595659	1.6188	5.42E-06	0.017001	-//-
111123064	26.49478969	105.8034765	1.9976	4.02E-06	0.015155	sp|P55906|BGH3_BOVIN Transforming growth factor-beta-induced protein ig-h3 OS=Bos taurus OX=9913 GN=TGFBI PE=1 SV=2//1.51711e-13
111128774	12.01024384	89.7447449	2.9016	3.58E-08	0.000224	sp|Q9UHN6|TMEM2_HUMAN Cell surface hyaluronidase OS=Homo sapiens OX=9606 GN=TMEM2 PE=1 SV=1//8.47923e-104
111138467	422.1143669	1076.218799	1.3503	2.08E-06	0.009791	-//-
Downregulated						
NONE						
**EE versus EA**						
Gene_id	readcount_EE	readcount_EA	log2FoldChange	pval	padj	Blast swiss prot
Upregulated						
111114597	64.45414549	164.5642875	1.3523	1.10E-05	0.022421	sp|Q05825|ATPB_DROME ATP synthase subunit beta, mitochondrial OS=Drosophila melanogaster OX=7227 GN=ATPsynbeta PE=1 SV=3//0
111134779	95.96920963	220.146285	1.1978	4.63E-06	0.010763	sp|Q96RW7|HMCN1_HUMAN Hemicentin-1 OS=Homo sapiens OX=9606 GN=HMCN1 PE=1 SV=2//1.53322e-44
111137234	47.33366626	190.8708725	2.0117	4.82E-10	7.84E-06	sp|B3EWZ3|CADN_ACRMI Coadhesin (Fragment) OS=Acropora millepora OX=45264 PE=1 SV=1//3.0659e-27

111137486	1453.663328	2320.429939	0.6747	3.63E-06	0.009849	sp|P11833|TBB_PARLI Tubulin beta chain OS=Paracentrotus lividus OX=7656 PE=2 SV=1//0
Downregulated						
111134528	579.5548922	309.704688	-0.90405	2.30E-06	0.007499	sp|Q5VYJ5|MALR1_HUMAN MAM and LDL-receptor class A domain-containing protein 1 OS=Homo sapiens OX=9606 GN=MALRD1 PE=1 SV=4//3.73958e-12
**AE versus EA**						
Gene_id	readcount_AE	readcount_EA	log2FoldChange	pval	padj	Blast swiss prot
Upregulated						
NONE						
Downregulated						
111113165	389.3810338	141.1739869	-1.4637	4.77E-07	0.008519	sp|P10079|FBP1_STRPU Fibropellin-1 OS=Strongylocentrotus purpuratus OX=7668 GN=EGF1 PE=1 SV=2//3.1654e-07

Gene Ontology Analysis

A comparison of significant GO terms enriched between AA and EE yielded one term broadly related to molecular function and two terms related to biological processes, all three of which were upregulated in EE (Supplementary data Table S3). The GO term with molecular function was found to be involved in arabinosyltransferase activity (hypergeometric test, $p=0.00063$). The GO terms involved in biological processes were found to function in actinobacterium-type cell wall biogenesis (hypergeometric test, $p=0.00063$) and peptidoglycan-based cell wall biogenesis (hypergeometric test, $p=0.048$). There were no significantly over-represented GO terms for all other comparisons (hypergeometric test, $p>0.05$) (AA vs AE, EE vs EA, or AE vs EA).

Table S5. Over-represented gene ontology terms

**AA versus EE**					
GO_accession	Description	Term Type	Over Represented pValue	Corrected pValue	Number of DEGs
Upregulated					
GO:0052636	arabinosyltransferase activity	molecular_function	2.62E-07	0.00062696	2
GO:0071766	Actinobacterium-type cell wall biogenesis	biological_process	2.62E-07	0.00062696	2
GO:0009273	peptidoglycan-based cell wall biogenesis	biological_process	2.99E-05	0.047728	2
Downregulated					
NONE					
**AA versus AE**					
NONE					
**EE versus EA**					
NONE					
**AE versus EA**					
NONE					

KEGG Pathway Enrichment Analysis of DEGs

Several pathways were significantly enriched in the DEGs for all treatment comparisons. A comparison of KEGG pathways enriched between AA and EE yielded 3 significantly enriched pathways: ribosome biogenesis in eukaryotes, phagosome and oxidative phosphorylation. The former was upregulated in EE and the two latter pathways were downregulated. A comparison of KEGG pathways enriched between EE and EA yielded 2 significantly enriched, upregulated pathways: phagosome and oxidative phosphorylation. Between AA and AE KEGG pathway enrichment analysis yielded 2 significantly upregulated, enriched pathways: dorso-ventral axis formation and notch signaling pathway. A comparison of KEGG pathways enriched between AE and EA also yielded 2 significantly enriched, upregulated pathways: dorso-ventral axis formation and notch signaling pathway. For the latter two comparisons (AA vs AE and AE vs EA), the genes that related to those two specific pathways, dorso-ventral axis formation and notch signaling pathway, were the same gene (Gene ID 111113165).

Table S6. Enriched pathways in the reciprocal transplant experiment.

**AA versus EE**							
Term	ID	Input number	Background number	P-Value	Corrected P-Value	Input	KEGG_ID/KO
Upregulated							
Ribosome biogenesis in eukaryotes	crg03008	1	76	0.044596	0.044596119	111125413	crg:105338454
Downregulated							
Oxidative phosphorylation	crg00190	2	97	0.002281	0.021542927	111138187, 111114597	crg:105329525, crg:105319097
Phagosome	crg04145	2	135	0.004309	0.021542927	111125973, 111137486	crg:105335713, crg:105343071
**AA versus AE**							
Term	ID	Input number	Background number	P-Value	Corrected P-Value	Input	KEGG_ID/KO
Upregulated							
Dorso-ventral axis formation	crg04320	1	58	0.010977	0.014121571	111113165	crg:105334163
Notch signaling pathway	crg04330	1	75	0.014122	0.014121571	111113165	crg:105334163
**EE versus EA**							
Term	ID	Input number	Background number	P-Value	Corrected P-Value	Input	KEGG_ID/KO
Upregulated							

Oxidative phosphorylation	crg00190	1	97	0.012156	0.025259856	111114597	crg:105319097
Phagosome	crg04145	1	135	0.01684	0.025259856	111137486	crg:105343071
**AE versus EA**							
Term	ID	Input number	Background number	P-Value	Corrected P-Value	Input	KEGG_ID/KO
Downregulated							
Dorso-ventral axis formation	crg04320	1	58	0.001838	0.002367749	111113165	crg:105334163
Notch signaling pathway	crg04330	1	75	0.002368	0.002367749	111113165	crg:105334163
